# The Need for Outreach in Preventing Suicide among Young Veterans

**DOI:** 10.1371/journal.pmed.1000035

**Published:** 2009-03-03

**Authors:** Jitender Sareen, Shay-Lee Belik

## Abstract

Jitender Sareen and Shay-Lee Belik discuss the implications of a new study showing an increased suicide risk in young men who left the UK Armed Forces.

There has been increasing concern about suicide in active service members. In the US military, suicide is the second most common cause of death [1]. Although mental health problems have clearly been associated with deployment to combat and peacekeeping operations [2,3], the literature on suicide has been mixed. Some studies in armed forces have found that the rate of completed suicide was lower than in the general population [4], while other studies have found higher rates [5] or no difference in suicide rates [6,7].

In this issue of *PLoS Medicine*, Navneet Kapur and colleagues examine the suicide rate in a very large sample of 233,803 individuals who left the UK Armed Forces, and in doing so they significantly advance our knowledge of suicide among veterans [8]. Unique features of this study include comparison of soldiers who had left the Armed Forces to those who were still serving and to the general population, as well as using administrative data to examine mental health service use by individuals prior to committing suicide.

Kapur and colleagues' study had a number of important findings. Although the overall prevalence of suicide was not higher among members who had left the Armed Forces, the risk of suicide among male soldiers less than 24 years of age was two to three times higher than among the general population and those who were currently serving. This study also showed that soldiers with short lengths of service (i.e., less than two years) were at increased risk of suicide compared to those with longer service periods.

## Limitations of the Study

These findings should be interpreted within the context of a number of limitations of the study. First of all, it is important to note that the overall prevalence of suicide in soldiers was very low. Among the 233,803 soldiers who had left the Armed Forces, there were 224 suicides (a prevalence of 0.096%). Thus, although the findings in the study are novel and important, they are based on a relatively small sample size of completed suicides and require further replication. Second, the present findings in the UK Armed Forces may not be generalizable to other countries, where there may be differences in selection of soldiers and in accessibility to health services post-discharge from the military. Third, although the rate of suicide was higher among young males who had left the Armed Forces than among the general population, it remains unclear whether being in the Armed Forces had any specific relationship to suicide. For example, it remains unknown whether the suicides were specifically associated with deployment-related mental health problems or physical injury during service.

Finally, there is a lack of detailed information about the specific psychopathology and life events of individuals completing suicide. In the suicide literature, the most accepted method of retrieving diagnostic information of completed suicide cases is psychological autopsy, in which those who knew the deceased are interviewed to try and reconstruct the deceased's thoughts, emotions, and actions [9]. The interviews used are specially designed to allow proxy-based diagnostic assessments. To the best of our knowledge, only one study, by Albert Wong and colleagues, has used this method to assess suicide in the military (Wong and colleagues studied suicides in Canadian soldiers assigned to UN peacekeeping duties) [6]. Further studies in the armed forces should consider using the psychological autopsy method to garner a more detailed understanding of suicide risk factors.

**Linked Research Article**

This Perspective discusses the following new study published in *PLoS Medicine*:

Kapur N, While D, Blatchley N, Bray I, Harrison K (2009) Suicide after leaving the UK Armed Forces—A cohort study. PLoS Med 6(3): e1000026. doi:10.1371/journal.pmed.1000026

Navneet Kapur and colleagues find that young men who leave the United Kingdom Armed Forces are at increased risk of suicide.

## Implications for Suicide Prevention

Kapur and colleagues' study is specifically useful for the UK Armed Forces in developing a suicide prevention strategy for those at highest risk for suicide. It provides evidence that young males with short length of service need to be targeted in screening and suicide prevention programs. Considering the original work of Émile Durkheim [10], one of the main causes of suicide from a social perspective is that the individual committing suicide has lost a sense of connection with society. Thus, the actively suicidal person who may be feeling disconnected from society is unlikely to make significant efforts to seek out mental health care. In addition, mental health problems are clearly a major risk for suicide, and recent data from US and Canadian military surveys show that most service members suffering from a mental disorder do not receive care [11,12]. Similarly, Kapur and colleagues found that only one in five of the ex-military personnel who committed suicide had any contact with specialist mental health care.

To address the challenge of preventing suicide due to some of the key factors noted above, a recent comprehensive review of general suicide prevention strategies underscores the need for a public health approach for suicide prevention [12]. This review suggests that suicide prevention should involve five major areas of prevention: (1) education and awareness programs for the general public and professionals; (2) screening methods for high-risk persons; (3) treatment of psychiatric disorders; (4) restricting access to lethal means; and (5) safe media reporting of suicide. Even as Kapur and colleagues' report is published, media might want to be careful not to sensationalize the findings. There is growing evidence suggesting that sensational stories of suicide increase the likelihood of copycat suicides, especially in youth [13]. To minimize the risk of copycat suicides, the US Centers for Disease Control has specifically developed guidelines on safe reporting of suicides by the media (http://www.cdc.gov/mmwr/preview/mmwrhtml/00031539.htm).

**Box 1: The 11 Initiatives of the US Air Force Suicide Prevention Program**

1. Leadership involvement in the spectrum of community-based suicide initiatives.
2. Addressing suicide prevention through professional military education.
3. Guidelines for commanders on how and when to use mental health services.
4. Community preventive services, including updating reporting systems to track patient care and prevention services.
5. Annual suicide prevention training for all military and civilian employees.
6. Changes in policies to ensure that individuals under investigation for legal problems (who are at increased suicide risk) are assessed for suicide potential.
7. Trauma stress response to help personnel deal with their reactions to traumatic incidents such as terrorist attacks.
8. Integrated Delivery System and Community Action Information Board, which provide a forum for cross-organizational review and resolution of individual, family, installation, and community issues.
9. Limited Privilege Suicide Prevention Program, i.e., those at suicide risk are afforded increased confidentiality when seen by mental health professionals.
10. Integrated Delivery System Consultation Tool, administered upon the commander's request, which allows commanders to assess unit strengths and identify areas of vulnerability.
11. Suicide event surveillance system. Adapted from http://afspp.afms.mil/ and [14].

Finally, in response to a rise in the number of suicides, the US Air Force instituted a multi-layered suicide prevention program in the mid-1990s (http://afspp.afms.mil/), consisting of 11 initiatives aimed at reducing stigma and improving capacity of personnel to recognize risk factors (Box 1). This program is the most comprehensively evaluated suicide prevention program in any military sample [14]. In a seminal cohort study of the program, Kerry Knox and colleagues evaluated its impact on risk of suicide and other outcomes that share underlying risk factors (e.g., other violent outcomes) [14]. In comparison with the pre-intervention period, the intervention was associated with a 33% relative risk reduction in suicides. During the study period, there were also reductions in other outcomes such as homicides and spousal abuse. Similar broad suicide prevention strategies should be considered in the UK and other military forces.
